# *S*-allylmercapto-*N*-acetylcysteine protects against oxidative stress and extends lifespan in *Caenorhabditis elegans*

**DOI:** 10.1371/journal.pone.0194780

**Published:** 2018-03-26

**Authors:** Naphtali Savion, Amir Levine, Shlomo Kotev-Emeth, Ulrike Bening Abu-Shach, Limor Broday

**Affiliations:** 1 Department of Human Molecular Genetics and Biochemistry and Goldschleger Eye Research Institute, Tel Aviv University, Tel-Aviv, Israel; 2 Department of Cell and Developmental Biology, Sackler Faculty of Medicine, Tel Aviv University, Tel-Aviv, Israel; Alexandria University, EGYPT

## Abstract

*S*-allylmercapto-*N*-acetylcysteine (ASSNAC) was shown in our previous study to activate Nrf2-mediated processes and increase glutathione level and resistance to oxidative stress in cultured endothelial cells. In this study, we explored the antioxidant protective effect of ASSNAC in *Caenorhabditis elegans (C*. *elegans)*. Treatment of *gst-4* reporter strain (CL2166) with increasing concentrations of ASSNAC (0.2 to 20 mM) for 24 hours and with ASSNAC (10 mM) for various time periods demonstrated a significant concentration- and time-dependent increase in Glutathione S-transferase (GST) gene expression (up to 60-fold at 20 mM after 24 hours). In addition, ASSNAC (2 mM; 24 hours) treatment of *C*. *elegans* strains N2 (wild type strain), *gst-4* reporter (CL2166) and temperature sensitive sterile strain (CF512) significantly increased GST enzyme activity by 1.9-, 1.5- and 1.8-fold, respectively. ASSNAC (2.0 mM; 24 hours) increased the reduced glutathione content in N2 and CF512 strains by 5.9- and 4.9-fold, respectively. Exposure of *C*. *elegans* (N2 strain) to a lethal concentration of H_2_O_2_ (3.5 mM; 120 min) resulted in death of 88% of the nematodes while pretreatment with ASSNAC (24 hours) reduced nematodes death in a concentration-dependent manner down to 8% at 2.0 mM. *C*. *elegans* nematodes (strain CF512) cultured on agar plates containing ASSNAC (0.5 to 5.0 mM) demonstrated a significant increase in lifespan compared to control (mean lifespan 26.45 ± 0.64 versus 22.90 ± 0.59 days; log-rank p ≤ 0.001 at 2.0 mM) with a maximal lifespan of 40 versus 36 days. In conclusion, ASSNAC up-regulates the GST gene expression and enzyme activity as well as the glutathione content in *C*. *elegans* nematodes and thereby increases their resistance to oxidative stress and extends their lifespan.

## Introduction

Overproduction of reactive oxygen species (ROS) results in oxidative stress, shown to damage cellular structures, including membranes, lipids, proteins and DNA and thus, plays a central role in many human diseases and in aging [1–3]. To manage oxidative stress, cells possess antioxidant protection mechanisms, which primarily consist of classical antioxidant enzymes such as superoxide dismutase (SOD) and catalase as well as reduced glutathione (GSH) and phase II detoxifying enzymes, including glutamate-cysteine ligase (GCL), heme oxygenase-1 (HO-1) and glutathione S-transferase (GST) [4]. The most abundant natural cellular antioxidant is GSH, found in cells in milimolar concentrations (1–10 mM) and plays an essential role in maintaining the cellular redox state [5]. The protective effect of GSH is based on the generation of its oxidized form, glutathione disulphide (GSSG), which is reduced back to GSH by glutathione reductase to maintain a high cellular GSH/GSSG ratio [6,7]. The antioxidant cellular mechanism involving phase II detoxifying enzymes is regulated by the antioxidant response element (ARE), which is activated by the transcription factor nuclear factor erythroid 2 (NF-E2)-related factor-2 (Nrf2) [4,8]. Upon oxidation of free sulfhydryl groups, known as the Nrf2 sensor for oxidative or electrophilic stress, Nrf2 is activated, translocated into the nucleus, where it interacts with ARE, resulting in expression of phase II antioxidants and detoxifying enzymes [8,9].

Oxidative stress and/or a low level of cellular GSH, the major intracellular redox buffer, are associated with the development and/or progression of numerous pathological conditions, such as, diabetes [10], neurodegenerative diseases [1], chronic inflammation [2], atherosclerosis and cardiovascular disease [2,3]. GSH deficiency involvement in the indicated pathologies has prompted several researchers to investigate new strategies for maintaining or restoring the GSH level. Therefore, it is important to find ways to activate Nrf2 and increase antioxidants cellular level, mainly GSH, which will prevent and/or minimize ROS-induced cellular damage. Indeed, the Nrf2 activator, dimethyl fumarate (Tecfidera®), which is already in clinical use for treating multiple sclerosis and sulforaphane, a natural dietary isothiocyanate found in broccoli, have been shown to induce phase II detoxification genes [11]. Further efforts to find new effective and safe Nrf2 activators suggest the group of allicin (active component in garlic)-derived thioallyl compounds as a candidates. Thus, Powolny et al. [12] have shown that the garlic constituent diallyl trisulfide increases *Caenorhabditis elegans* (*C*. *elegans*) lifespan via SKN-1 (the worm ortholog of the Nrf2) activation. A recent study further showed that the natural thioallyl compounds *S*-allylcysteine (SAC) and *S*-allylmercaptocysteine (SAMC) increase *C*. *elegans* oxidative stress resistance and extend their lifespan by modulating SKN-1/Nrf2 [13], while another study reported SAC antioxidant activity, however with no effect on lifespan in *C*. *elegans* [14].

In the present study, we explored the protective effect of another allicin derivative, the allicin conjugate with N-acetylcysteine (NAC)–*S*-allylmercapto-*N*-acetylcysteine (ASSNAC) [15], not yet studied in *C*. *elegans*. ASSNAC was shown to undergo a thiol-exchange reaction thereby oxidizing cellular free sulfhydryls, resulting in Nrf2 activation. Consequently, free NAC is released and is utilized as a substrate for glutathione biosynthesis [15]. Thus, ASSNAC plays a dual role, activating Nrf2 and supplying cysteine. More than that, the highly hydrophobic nature of the s-allyl mercaptan moiety facilitates ASSNAC cellular penetration. Indeed, we demonstrated the ability of ASSNAC to increase expression of phase II detoxifying enzymes and glutathione level in cultured vascular endothelial cells, thus increasing the cellular protection against oxidative stress [15] and attenuating clinical symptoms of experimental autoimmune encephalomyelitis (EAE), the animal model of multiple sclerosis [16]. Our current study explores the ability of ASSNAC to increase glutathione content, GST gene expression and enzyme activity in *C*. *elegans*, which protect them against H_2_O_2_-induced death and extend their lifespan.

## Materials and methods

### *S*-allylmercapto-*N*-acetylcysteine (ASSNAC)

ASSNAC was synthetized as previously described [15] and a preparation with purity of 96.8% was used in this study. For preparation of ASSNAC stock solution, 100 mg of ASSNAC was dissolved in 8.48 ml DDW plus 2.12 ml Na_3_PO_4_ (0.2 M) to a final concentration of 40 mM in phosphate buffer (40 mM; pH = 7.4).

### *C*. *elegans* strains and growth conditions

*C*. *elegans* is a well-accepted animal model for exploration of antioxidant compounds and their effect on lifespan [17,18]. *C*. *elegans* strains used were N2 wild-type (WT; Bristol isolated), CF512 (*fer-15(b26) II;fem- 1(hc17) IV*) [19] and CL2166 (*dvIs19[pAF15(gst-4p*::*GFP*::*NLS)]*) [20]. All strains were grown in nematode growth medium (NGM) supplemented with *E*. *coli* strain OP50 and except for strain CF512 were maintained at 20°C [21]. *C*. *elegans* gravid adults were hypochlorite-bleached and assays were performed on synchronized L4 stage animals. Lifespan assays were performed on NGM agar plates using the CF512 strain, an established strain for lifespan experiments [22–24]. Biochemical assays were performed on cultured nematodes in liquid M9 medium supplemented with OP50 (3 μl/ml), cholesterol (5 μg/ml, penicillin (100 U/ml), streptomycin (0.1 mg/ml) and nystatin (12.5 U/ml).

### GST expression determination

GST induction assays were performed using the GFP reporter strain CL2166 (*dvIs19[pAF15(gst-4p*::*GFP*::*NLS*)]). The reporter function was verified by nematodes exposure to 3 mM hydrogen peroxide to induce oxidative stress, which is known as a positive GST inducer [20]. ASSNAC effect was studied using developmental stage synchronized nematodes. After bleaching, L1 arrested nematodes were dropped on NGM agar plates for 48 hours. The nematodes reached mid-L4, at which point they were collected and washed with M9 buffer by centrifugation (1.5 min; 2500 RPM), divided into Erlenmeyer flasks, at a density of 1000/ml and treated with ASSNAC at 20°C. In all treatments lasting longer than 12 hours, OP50 was added to the cultures in order to prevent starvation. At the end of treatment, nematodes were dropped on empty NGM plates, allowed to dry and 50–100 nematodes were picked onto slides coated with agar containing 3 μL of 0.1% tricaine and 0.01% tetramisole in M9. Slides were examined by a Nikon 80i microscope and GFP fluorescence was captured using a Nikon DS-U1 camera. Two images were captured for each nematode, bright-field (to view the animals’ morphology) and fluorescent. To quantify the intensity of fluorescence, the two images were stacked using ImageJ (https://imagej.nih.gov/ij/) and based on each nematode’s morphology, areas of the pharynx, the pharyngeal-intestinal valve and the most anterior intestinal rings were selected for analysis.

### GST activity determination

Nematodes were washed in M9 and centrifuged (3 cycles; 1.5 min; 2500 RPM), dispersed in 0.2 ml Tris-HCl buffer (50 mM; pH = 7.5) with EDTA (5 mM), lysed by freezing in liquid nitrogen and immediate thawing at 37°C (3 cycles), sonicated at 4°C, centrifuged (10 min; 15,000Xg; 4°C) and supernatants collected. GST activity was determined using Glutathione S-Transferase assay kit (Sigma CS0410, Sigma Aldrich, St. Louis, MO). Assay reaction product was placed in 1 ml quartz cuvettes and absorbance at 340 nm was recorded by a spectrophotometer (Shimadzu UV-2450, Kyoto, Japan). Supernatant protein content was determined by the Lowry method [25]. GST activity values are presented as μmole/min/mg protein.

### Glutathione determination

Nematodes were collected by centrifugation (1.5 min; 2500 RPM) and washed 3 times in phosphate buffered saline. Pellets were lysed in HCl (10 mM) containing EDTA (5 mM) by three cycles of liquid nitrogen freezing and thawing. Proteins were precipitated by addition of 5-sulfosalicylic acid (SSA; 10%) followed by centrifugation (13,000Xg; 5 min) and supernatants and pellets were collected for glutathione and protein determination, respectively. Anderson recycling method [26] was used to determine total glutathione (GSH + GSSG). To determine GSSG, 2-vinylpyridine was used to conjugate GSH and remove it from the mixture as described [26]. The following reagents used in this assay were purchased from Sigma (St. Louis, MO, USA): 5,5'-dithiobis(2-nitrobenzoic acid) (DTNB), SSA, GSSG, b-nicotinamide adenine dinucleotide 2'-phosphate reduced tetrasodium salt (NADPH), glutathione reductase and 2-vinylpyridine. Protein pellets were lysed in 0.5 N NaOH and quantified by the Lowry method [25]. Glutathione values (GSH equivalent) were calculated and presented as nmole/mg protein.

### Exposure to oxidative stress and survival determination

Nematodes were synchronized by bleaching and grown for 2 days in Erlenmeyer flasks in a liquid medium at a density of 2500 worms/ml at 20°C to larval stage mid L3. Then, nematodes were pretreated with ASSNAC for 24 hours at 20°C, centrifuged (1.5 min; 2500 RPM), washed three times with M9 buffer and further treated with or without H_2_O_2_ (120 min at 20°C; 3.5 mM) in M9 as described [27]. Around 100 nematodes from each treatment were dropped on NGM plates (containing OP50), allowed to dry for 30 min and the number of live and dead nematodes was counted. A platinum wire was used to gently touch the animals to determine if they are alive. Plates with treated worms were stored at 20°C for 24 hours. This recovery period allowed distinguishing between dead and live paralyzed animals.

### Lifespan determination

*C*. *elegans* strain CF512 was grown on NGM agar plates with OP50 at 15°C. Nematodes were bleached and eggs were dropped on NGM agar plates containing OP50 and grown for 3 days at 25°C until day 1 of adulthood.

For the lifespan assay, plates were supplemented with phosphate buffer (control; 0.2 ml; 40 mM; pH = 7.4) or ASSNAC (0.2 ml) and incubated for 24 hours for absorption into the agar to final ASSNAC concentrations of 0.5, 2 and 5 mM. OP50 was added and plates were incubated for additional 24 hours. Adult nematodes (20 per plate, 6 plates per treatment) were transferred to these plates and grown at 20°C. Nematodes were transferred twice a week to control or ASSNAC-treated fresh plates, respectively and their survival was monitored three times a week. Animals were scored at the indicated times and considered dead upon failure to respond to touch. Missing animals were censored on day of loss. Survival results were plotted and statistically analyzed by OASIS (Online Application for the Survival Analysis; http://sbi.postech.ac.kr/oasis/surv) [28].

### Statistical analysis

The significance of the results was tested using the following tests: Kruskal-Wallis Non-Parametric 1-Way ANOVA, 2-Way ANOVA with Bonferroni post hoc test and by OASIS [28]. Differences of p≤0.05 were considered significant.

## Results

### ASSNAC effect on GST expression and activity

**Fig 1:**
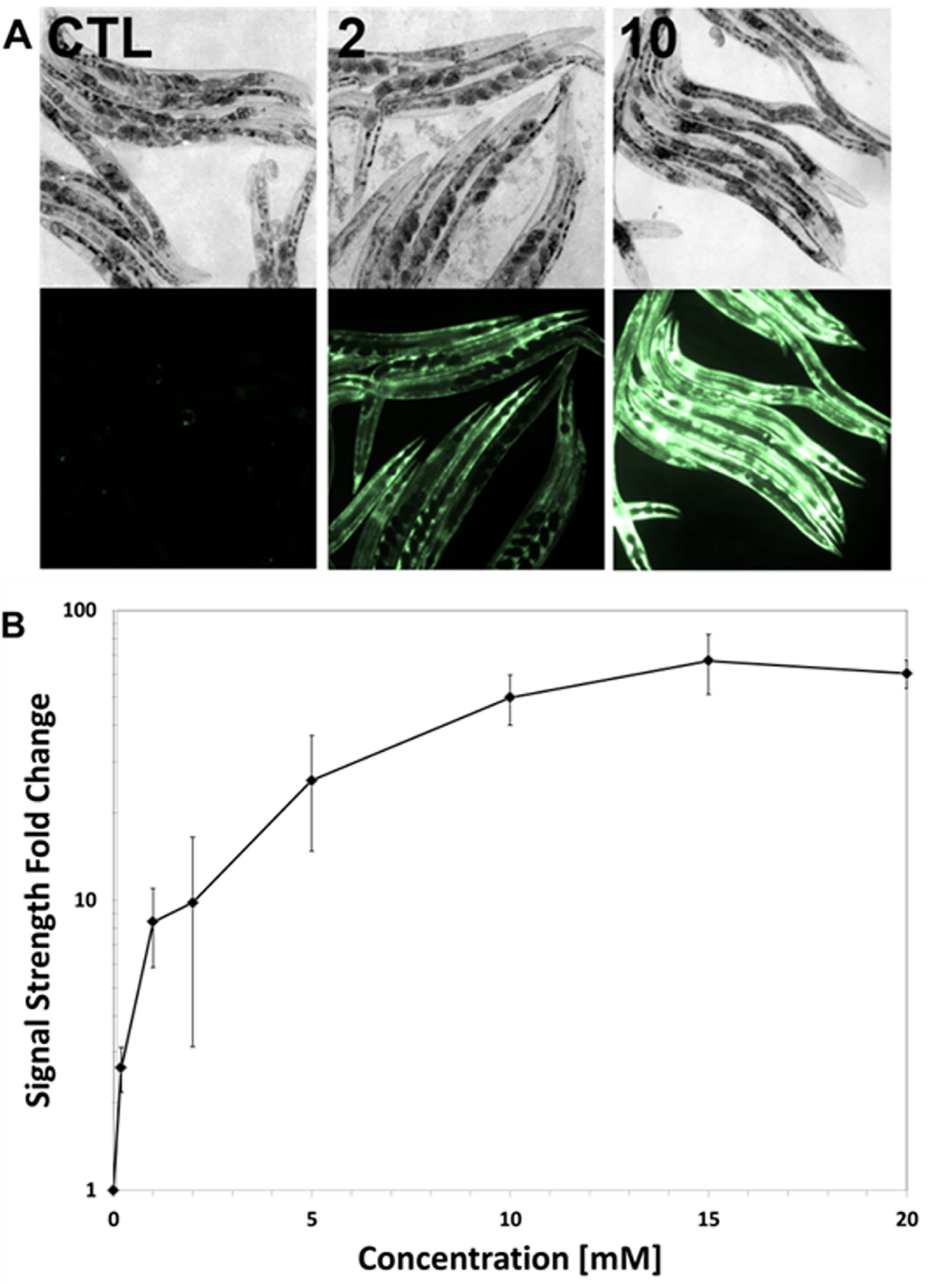
Concentration-dependent effect of ASSNAC on *GST-4p*::*GFP* induction. *C*. *elegans* CL2166 strain expressing the reporter gene *gst-4p*::*GFP* was cultured in suspension in the presence of increasing concentrations of ASSNAC for 24 hours. Fifty to hundred nematodes were picked onto slides and the GFP fluorescence intensity was captured. **A.** Images of the nematodes were taken under bright-field (upper panel) and fluorescent light (lower panel) [control (CTL), 2 and 10 mM ASSNAC]. **B.** Intensity of the fluorescent signal was quantitated and presented as mean ± S.D. of three independent experiments. GST expression in ASSNAC-treated nematodes was found to be statistically different from control as determined by Kruskal-Wallis Non-Parametric 1-Way ANOVA (p≤0.003).

**Fig 2:**
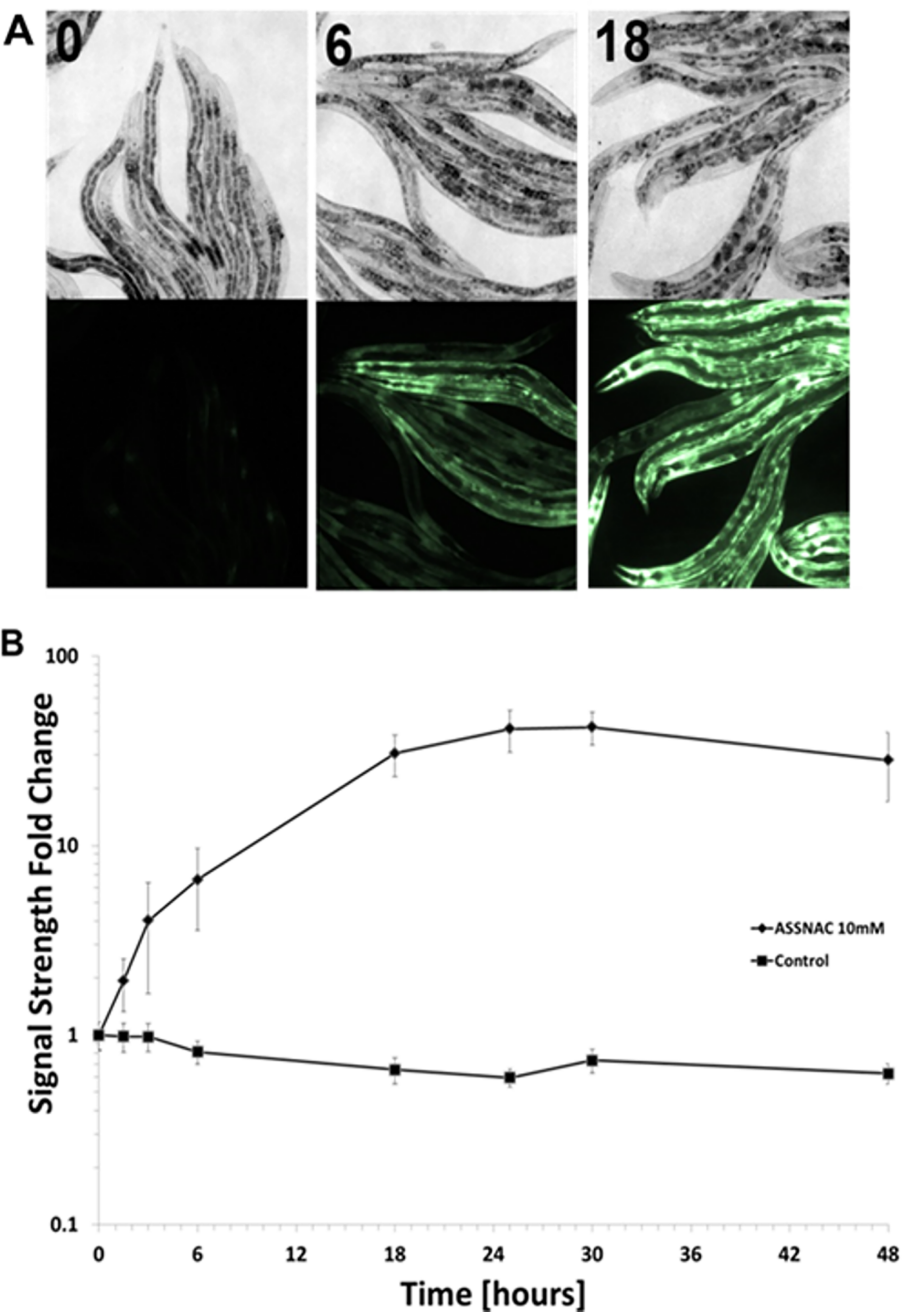
Time-dependent effect of ASSNAC on *GST-4p*::*GFP* induction. *C*. *elegans* CL2166 strain expressing the reporter gene *gst-4p*::*GFP* was cultured in suspension in the absence (control) or presence of ASSNAC (10 mM) for the indicated time points and 50–100 nematodes were picked onto slides and analyzed as described in Fig 1. **A.** Bright-field (upper panel) and fluorescent light (lower panel) images are presented (0, 6 and 18 hours). **B.** The intensity of the fluorescent signal was quantitated and presented as mean ± S.D. of three independent experiments.

*C*. *elegans* (strain CL2166 with *gst-4p*::*GFP* reporter) cultured in growth medium and treated with ASSNAC (0.2 to 20 mM; 24 hours) demonstrated a significant concentration-dependent increase in the fluorescent GFP, representing GST expression, reaching close to plateau at a concentration of 10 mM with a maximal increase of 60-fold (Fig 1). Induction of fluorescent GFP by ASSNAC (10 mM) was time-dependent, starting within the first 90 min of treatment and reaching a maximal effect after 24 hours with a slight decrease in signal strength after 48 hours (Fig 2).

**Fig 3:**
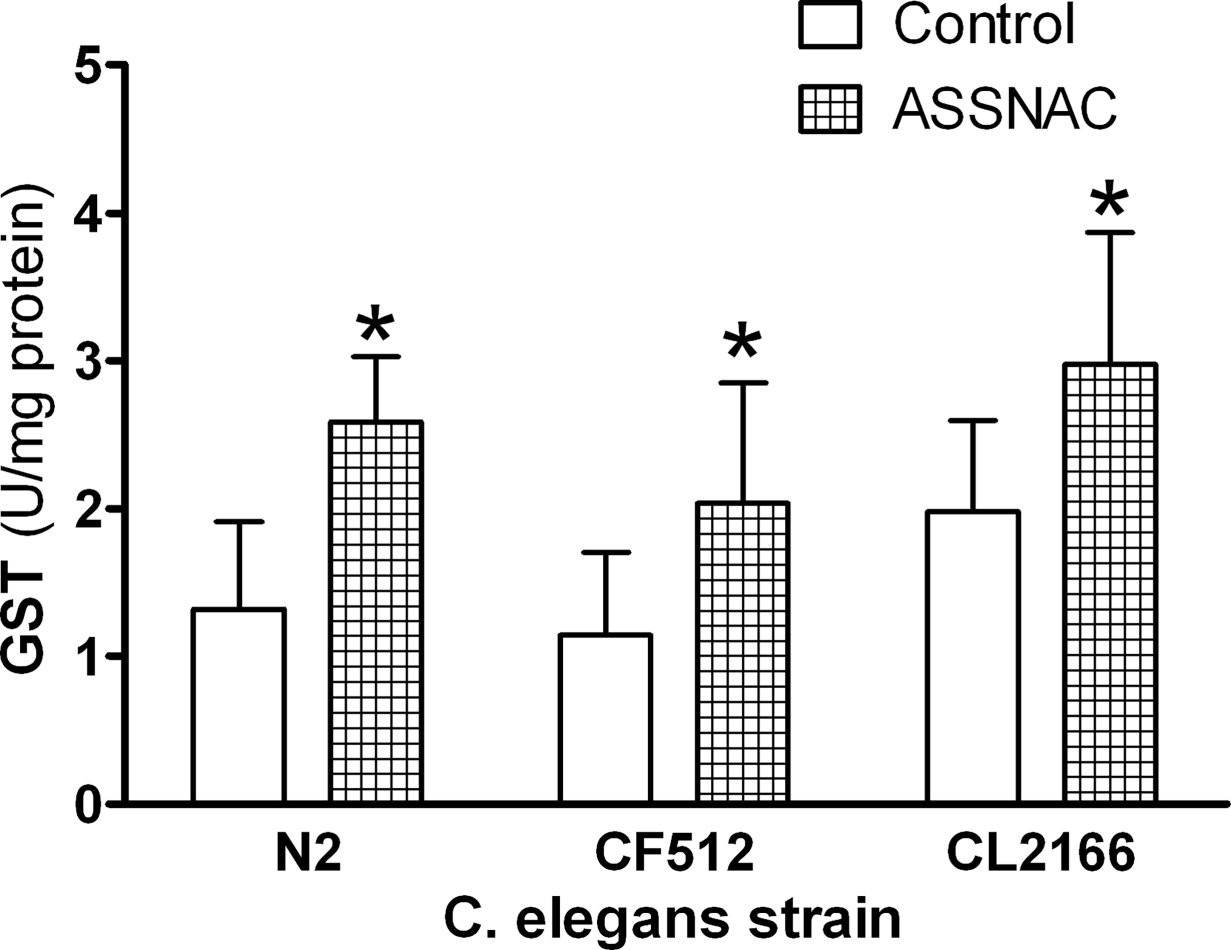
Effect of ASSNAC on GST enzyme activity. *C*. *elegans* strains N2, CF512 and CL2166 were cultured in suspension and treated without (control) or with ASSNAC (2.0 mM for 24 h). Nematodes were collected, lysed and GST activity was determined and presented in Units (μmole/min; mean ± S.D.). GST activity in ASSNAC-treated nematodes was found to be statistically different from control as determined by 2-Way ANOVA with Bonferroni post hoc test (*p≤0.001).

GST enzyme activity was studied in *C*. *elegans* treated with or without ASSNAC (2 mM; 24 hours). ASSNAC induced GST activity in *C*. *elegans* strains N2, CF512 and CL2166 by 1.95-, 1.78- and 1.51-fold, respectively (Fig 3).

### Glutathione up-regulation by ASSNAC

**Fig 4:**
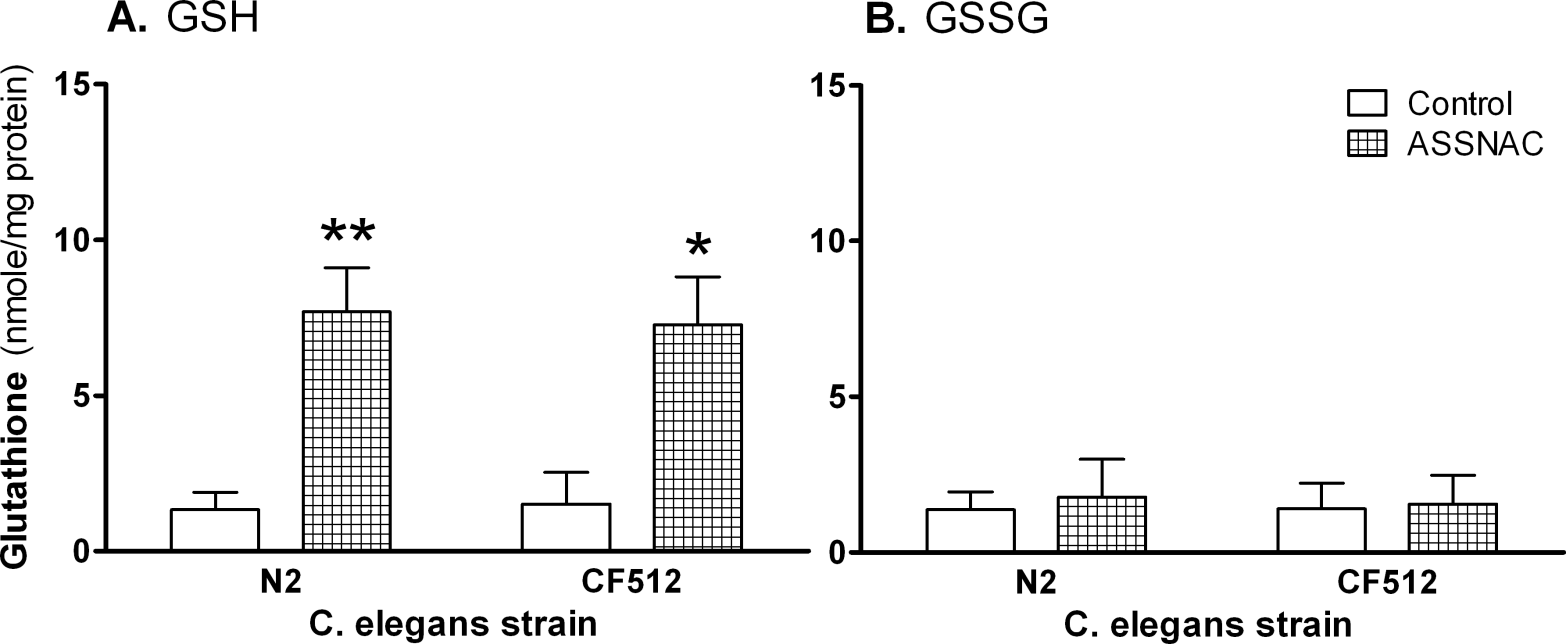
ASSNAC effect on glutathione level. *C*. *elegans* (strains N2 and CF512) cultured in suspension were treated without (control) or with ASSNAC (2.0 mM for 24 h). Nematodes were collected and both GSH (Reduced) and GSSG (Oxidized) glutathione were determined. Results are presented as mean ± S.D of three independent experiments. The increase in GSH level in ASSNAC-treated nematodes was found to be statistically significant as determined by 2-Way ANOVA with Bonferroni post hoc test (*p≤0.01; **p≤0.001).

GSH represents 50% of the total glutathione in control non-treated *C*. *elegans* (strains N2 and CF512). Following ASSNAC treatment (2 mM; 24 hours), a significant increase in nematodes GSH content (by 5.9- and 4.9-fold, respectively) was observed, while the level of oxidized glutathione (GSSG) was not affected (Fig 4).

### ASSNAC protection from hydrogen peroxide cytotoxicity

**Fig 5:**
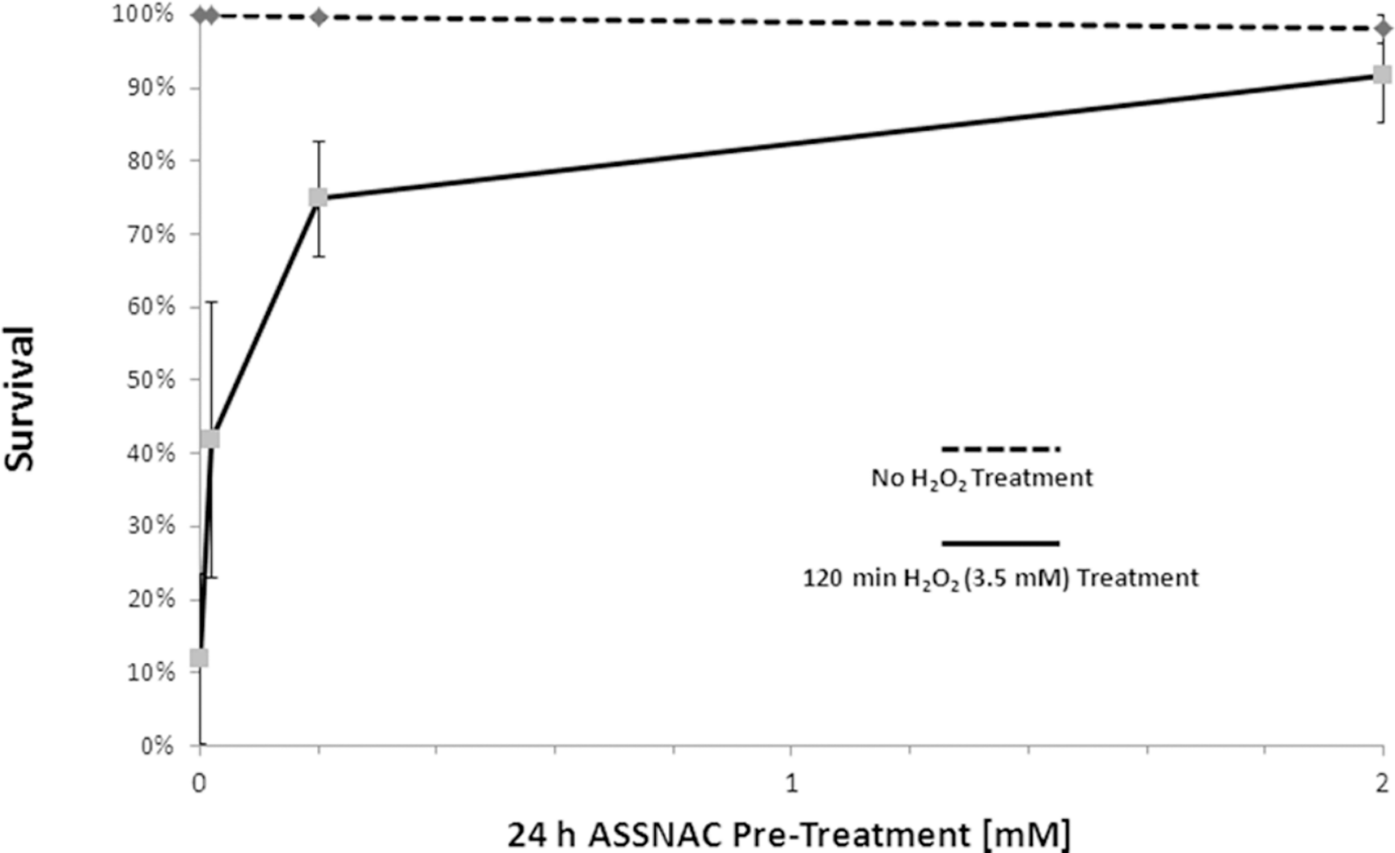
ASSNAC protective effect against oxidative stress. *C*. *elegans* (N2 strain) in suspension was pretreated with ASSNAC (0.02 to 2.0 mM for 24 hours) and not exposed (control) or exposed to H_2_O_2_ (3.5 mM for 2 hours). Ninety to 180 nematodes were examined and the percentage of survival is presented as mean ± S.D. of three independent experiments. Percentage of survival in ASSNAC-treated nematodes was found to be significantly different from that in control (p≤0.02) as determined by Kruskal-Wallis Non-Parametric 1-Way ANOVA.

Exposure of *C*. *elegans* (N2 strain) in growth medium to a lethal concentration of H_2_O_2_ (3.5 mM; 120 min) resulted in death of 88% of the nematodes. Pretreatment with increasing concentrations of ASSNAC (0.02 to 2.0 mM) for 24 hours prior to exposure to H_2_O_2_ significantly reduced nematodes death. A concentration-dependent increase in the survival rates was observed, reaching close to complete protection (92% survival) at 2.0 mM (Fig 5).

### ASSNAC effect on *C*. *elegans* lifespan

**Fig 6:**
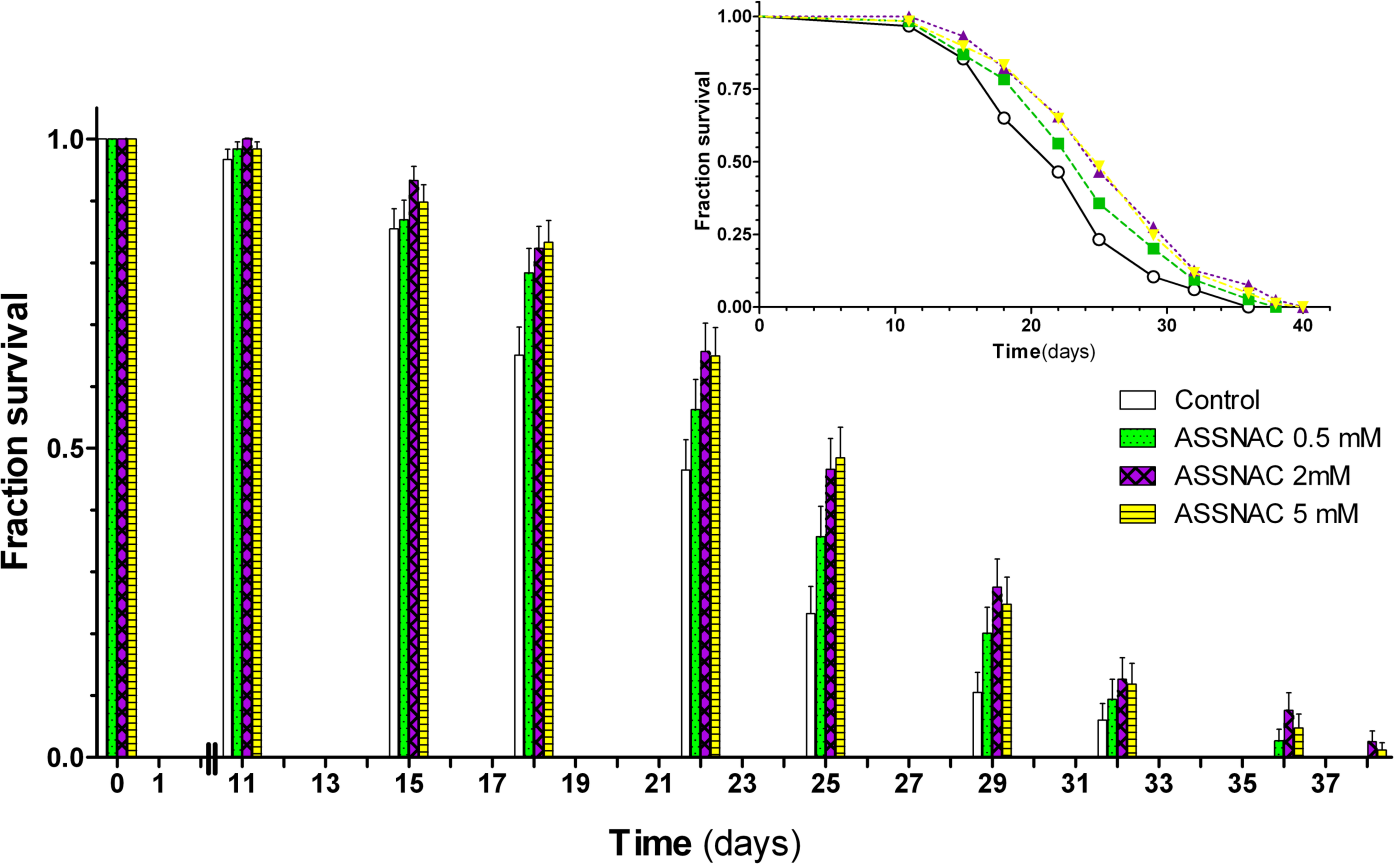
ASSNAC-induced extension of *C*. *elegans* lifespan. Adult synchronized L4 stage *C*. *elegans* (strain CF512) nematodes (20 per plate, 6 plates per treatment) were maintained on NGM agar plates containing ASSNAC (final concentrations: 0.5, 2 and 5 mM) or vehicle (phosphate buffer) and supplemented with OP50 at 20°C. Nematodes were transferred to fresh plates twice a week and their survival was monitored three times a week and presented as fraction of survival (mean ± SE of 6 plates). Experiment was repeated twice and one experiment is presented.

In order to study the effect of ASSNAC on lifespan, *C*. *elegans* nematodes (strain CF512) were cultured on agar plates containing ASSNAC (0, 0.5, 2.0 and 5.0 mM). Exposure to ASSNAC resulted in a significant dose-dependent increase in lifespan with a maximal effect at 2.0 mM. Mean lifespan was increased in treated cultures to 26.45 ± 0.64 days compared to 22.90 ± 0.59 days in control; log-rank p ≤ 0.001 with maximal lifespan of 40 versus 36 days, respectively (Fig 6 and Table 1).

**Table 1:** ASSNAC effect on lifespan.

Treatment	Mean Lifespan ± S.E. (Days)	Change of Mean Lifespan	Log-rank test
Phosphate buffer	22.90 ± 0.59	-	-
ASSNAC 0.5 mM	24.82 ± 0.63	1.08	*p<0*.*0290*
ASSNAC 2 mM	26.45 ± 0.64	1.16	*p<0*.*0001*
ASSNAC 5 mM	26.16 ± 0.63	1.14	*p<0*.*0002*

Results of the experiment described in Fig 6 were statistically analyzed by OASIS [28] and the mean lifespan ± S.E. and the significance (*p* value) are presented.

## Discussion

Age-related degeneration diseases and aging are believed to be the result of oxidative stress (ROS activity). Exposure to ROS, created through normal cellular metabolism and environmental hazards and inflammation, results in damage to cellular structures, leading to loss of critical cell functions [29]. A current established whole animal model for the study of aging and longevity is *C*. *elegans* because of its relatively short lifespan and the well-defined genetic and environmental factors that affect it [30–33]. *C*. *elegans* transcription factor SKN-1 (the ortholog of the mammalian Nrf2) was shown to induce a phase II detoxification response, which protects against oxidative stress and thus, is active in multiple longevity pathways [34,35]. Previous studies suggested that lifespan may be extended by enhanced resistance to oxidative stress [36] and indeed, herb mixture KPG-7 that contains several components exhibiting antioxidant activity has been shown to extend the lifespan of *C*. *elegans* [37]. Therefore, in the present study we explored the capacity of ASSNAC to increase the antioxidative activity in *C*. *elegans* and thus, to extend its lifespan. Indeed, our study demonstrates the capacity of ASSNAC to increase the expression and activity of the antioxidant enzyme GST in a time- and concentration-dependent manner. The fact that this enzyme is under strict regulation of SKN-1 may indicate that ASSNAC functions in *C*. *elegans* via SKN-1 activation, similarly to our previously observed ASSNAC-induced Nrf2 activation in endothelial cells [15]. Assessment of GST expression kinetics during ASSNAC treatment demonstrates maximal GST expression at a concentration of 10 mM within 24 hours of treatment. However, up to 75% survival is already achieved at a concentration of 0.2 mM, while only a partial increase of 2.6-fold in GST expression is observed at that concentration, suggesting that the further significant increase in GST expression up to 60-fold at higher ASSNAC concentrations has no further clear effect on nematodes survival.

GSH is the most abundant non-protein thiol in mammalian cells, acting as a major reducing agent by maintaining a tight control of redox status and thus, serving as a cellular antioxidant [38,39]. ASSNAC elevated nematode GSH content and thereby protected them from oxidative stress as indicated by their increased resistance to H_2_O_2_-induced cytotoxicity. This result is complementary to the recent observation that a modest decrease in GSH level in young adult worms may promote their stress resistance and life span, whereas depletion of GSH is detrimental to freshly hatched and developing worms [40]. Our results are supported by a previous study [41], in which S-linolenoyl-glutathione was shown to elevate GSH level and thereby increase the stress resistance and thus, extend the lifespan of wild-type N2 *C*. *elegans* nematodes. Altogether, these data suggest that the increase in antioxidant resistance and the extended lifespan in *C*. *elegans* nematodes are a direct result of the ASSNAC-induced increase in GSH level.

The observed capacity of ASSNAC to extend lifespan in *C*. *elegans* is similar to the previously reported activity of other garlic-derived thioallyl compounds such as *S*-allylcysteine (SAC), *S*-allylmercaptocysteine (SAMC) [13] and diallyl trisulfide (DATS) [12] as well as that of curcumin [31]. These molecules are suggested to exert their antioxidative and lifespan extension properties via Nrf2 activation similarly to ASSNAC, however, unlike ASSNAC, they do not directly replenish the cysteine cellular level.

The results of the present study demonstrate a significant increase in mean lifespan, while only a slight but not significant increase in maximal longevity is observed, similarly to the results of previous studies using DATS [12] and other SKN-1 activators [42,43]. Taking into account that *C*. *elegans* is a well-established model for the study of oxidative stress-associated human diseases such as neurodegenerative diseases [44] suggests a significant potential for ASSNAC for the treatment of relevant complex human diseases.

In conclusion, the current study strengthens our observation that ASSNAC augments Nrf2-regulated genes expression and the glutathione level. Furthermore, it provides novel data demonstrating the capacity of ASSNAC to protect an entire organism from oxidative stress and extend its lifespan.
